# IVF in Sri Lanka: A concise history of regulatory impasse

**DOI:** 10.1016/j.rbms.2016.02.003

**Published:** 2016-03-11

**Authors:** Bob Simpson

**Affiliations:** Department of Anthropology, Durham University, Dawson Building, Lower Mountjoy, Stockton Road, Durham, UK

**Keywords:** Buddhism, Catholicism, IVF, regulation, Sri Lanka

## Abstract

This article outlines the development of IVF in Sri Lanka from the first successful births in the late 1990s and over the subsequent 15 years. It is based on anthropological fieldwork carried out at various points during this period. The piece focuses on the challenges entailed in achieving regulation of the new reproductive technologies against a backdrop of: (i) a bitter civil war; (ii) a complex mosaic of different religious traditions (specifically, Buddhism, Catholicism, Hinduism and Islam); and (iii) a shift towards neo-liberal marketization, particularly in relation to specialist and hi-tech medical interventions. The article concludes that ‘soft’ regulation operates both to avoid conflict around highly contentious issues in debates about reproductive rights as well as to enable commercially driven developments in technologically specialised areas of medicine.

## Introduction

Sri Lanka lies off the south-west coast of India. It is home to some 20 million people, the majority of whom are ethnically Sinhalese and Buddhist by religion (70%). A minority of Sinhalese are Christians. The Island also has a well-established Tamil minority (18.2%), who are made up of Hindus, Muslims and Christians, and smaller minorities of Malays and those of Euro-Asian descent known as Burghers (Department of Census and Statistics, 2014). Sadly, Sri Lanka became known in recent decades for the ethnic strife and bloodshed arising from the bitter secessionist struggle between the Government of Sri Lanka and the Liberation Tigers for Tamil Eelam (LTTE), who were fighting to establish an independent state in the North of the Island. The war began in the early 1980s and reached a bloody climax in 2009. Estimates vary, but the loss of life over the 25 years of the war was in the region of 80–100,000.

It was against this backdrop that in 2000 I began a project exploring the reception of new reproductive and genetic technologies. The precise locus of this work on IVF was not the community of users, nor the laboratories in which IVF was taking place, but the community of experts who were identified – sometimes by themselves and sometimes by others – as the ones who would debate and agree on issues, write documents, give advice, say what unfamiliar things were to mean and otherwise vernacularize the flow of challenging technological possibilities that were then becoming available to assist reproduction. In connection with this research I made a total of four visits between 2000 and 2003, each lasting between one and three months. These visits coincided with the ebb and flow of the war. The capital city Colombo was relatively safe at that time, and the tourist industry in the south of the Island continued largely oblivious to the mayhem that was happening in the north. Nevertheless, bombings and shootings did happen from time to time, and Colombo was heavily militarized, with checkpoints seemingly at every turn. The smiling, hospitable and easy-going persona that most Sri Lankans like to project was at odds with the anguish and anxiety that many were feeling as civil strife around them went from bad to worse. It might be thought odd that such an expensive, exclusive and demanding technology as IVF might be taking off in such challenging circumstances. At the time of my fieldwork, the challenge for regulators was how to make technologies that had infiltrated from outside into something that appeared to be owned from within, yet at the same time looked just like IVF delivery anywhere else in the world (in terms of standards, governance, ethics, operating procedures and protocols).

In this article I want to attempt what might be described as a concise history of regulatory impasse that captures the journey of this dazzling new technology from its introduction to the present day. What I am keen to illustrate is the practical tension that exists between regulatory strategies and the rationalities that underpin these on the one hand, and the evident facts of ethnic diversity and religious pluralism on the other. Significant in this regard was the fact that anxieties about national disintegration had brought reproduction, infertility and its treatment into the public gaze with an urgency and an edge that it might not have had in peace time; in symbolic terms, the state of reproduction was closely intertwined with the reproduction of the state (Simpson, 2004). The country's birth rate had been decreasing steadily over a number of years as a result of family limitation, migration and the war, and was set to drop further from 1.2% in 1998 to 1% for a number of years to follow (Laksman and Tisdell, 2002). Amidst a growing concern about a shrinking population, and particularly among the 70% of the population who were Sinhala Buddhists, IVF made its first appearance in Sri Lanka in the late 1990s. At that time it was a service supplied to elites and accessible only on the margins of a predominantly Colombo-based private sector. Nevertheless, its visibility was then high and its momentum strong. This was a very modern response to a problem that, in the fragile pronatalism of the time, many would understand and empathize with. In the midst of anxiety and a palpable despair at the way the war was eroding the quality of life and liberty, news of IVF-conceived babies signalled optimism, hope and a brighter future.

## The ‘first’ IVF child

The first IVF child on Sri Lankan soil was born in November 1999 to a Tamil couple from Batticaloa. The team of doctors was headed by Dr. V Arulandarajah, a UK-trained Tamil doctor who was Director of the ICSI Lanka Fertility Centre in Colombo. In the absence of appropriately trained local specialists, Dr. Arulandarajah had assembled a multinational team which was able to carry out an IVF procedure that resulted in the birth of a child by Caesarean section in a private hospital in Colombo. The birth was widely reported in the Sri Lankan press. The message was one of ‘miracles’ and ‘hope’. It was presented as a ‘first’ that would open the way to wider access to IVF in Sri Lanka. Whereas previously, couples seeking infertility treatment had to travel to India, Singapore or Europe, the provision of services locally would make access to IVF cheaper and therefore more widely available to Sri Lankans.

A much more widely reported ‘first’ occurred in July 2002 with the birth of a baby girl called Janaki. Throughout the extensive reporting of this birth a strong theme emerged. The team, led by Professor Harshalal Seneviratne, was all Sri Lankan and did not rely on foreign experts. This demonstration of technological self-sufficiency was cause for much pride. In contrast to the earlier IVF ‘first’, the manner of this conception was not tainted by dependency upon, or complicity with, outsiders. Although the team were not religiously partisan in their claims, the achievement resonated strongly with the nationalist sentiments and aspirations of the Buddhist majority community. The national press was not slow to celebrate the fact that it was the birth of a Sinhala Buddhist baby. In proclaiming her gratitude to reporters, the mother of the baby expressed her desire that ‘every doctor who helped me should become a (future) Buddha’. In other words, the doctors' work was not just medically beneficent but was also read as a meritorious act of such greatness that the highest possible rebirth should be the reward for their actions.

The Vindana Reproductive Health Centre, under the directorship of Professor Harshalal Seneviratne, quickly became Sri Lanka's premier IVF facility. However, in its early days another important figure in Sri Lanka's IVF story was Dr. Rohana Haththotuwa, the Vindana Centre's clinical co-ordinator. Keen to establish his own facility, he left in 2000 to establish the Ninewells CARE Mother and Baby Hospital. Although not part of the pioneering IVF team, he went on to establish a 30-bed facility that advertises a range of treatments, including IVF, aimed at giving women the hope of ‘safe and happy motherhood’.

In the early days of IVF, ICSI Lanka, Vindana and Ninewells were the main providers. Each of these facilities had its own particular link to specialists abroad who would provide technical support, advice and oversight. ICSI Lanka had close associations with the MultiCare team operating out of St George's Hospital in London, Vindana with Simon Fishel and CARE Fertility in Nottingham, and Ninewells with the Singapore-based Sri Lankan, Professor Arif Bongso, who was known for his pioneering work on intra-cytoplasmic sperm injection.

From these small beginings IVF gradually became more available to local couples facing infertility problems. The opening of these clinics also raised the possibility of Sri Lanka as a future destination for what has been problematically referred to as ‘reproductive tourism’ (Inhorn and Patrizio, 2009). In the early days, those seeking IVF from abroad were mostly ex-pat Sri Lankans wishing to come ‘home’ for treatment on the basis that it was not only cheaper but also offered the possibility of cultural and language familiarity and access to extended family support networks. The fledgling supply of IVF in Sri Lanka also supplied a small but steady stream of clients from the Maldives, who, lacking local facilities have long since used Sri Lanka as the nearest place where hi-tech treatments can be sourced.

## Slumbering sentinels

In March 2000, not long before my research started, Professor Jayasekara, the country's leading geneticist, had given a public lecture entitled ‘Genethics in Sri Lanka: The Slumbering Sentinels’. As both a geneticist and a Catholic he had been stirred by concerns about the unexamined and mostly unregulated spread of the new technologies in Sri Lanka. The invitation to give the lecture had come from the Sri Lanka Medical Association, and his audience was made up of a wide and influential group of professionals. In his lecture he provided an overview of the range of ethical, legal and social challenges. The ‘sentinels’ referred to in his title were law and human rights as these relate to medical science and technology. The image was one used in an earlier paper by Ranasinghe (1984), who in turn took it from the eminent Sri Lankan lawyer CG Weeramantry (1983). The message was clear: doctors, lawyers and philosophers, whose responsibility it is to watch over these developments, were in certain respects failing in their duties and responsibilities, and as a result basic human rights were falling into jeopardy. Professor Jayasekara's lecture was a spirited call for academics, the government and the public to ‘wake up’ and set about the task of devising workable strategies for how to frame, assimilate, regulate and, perhaps, resist powerful developments in western science, technology and research.

When contemplating the new genetic and reproductive technologies (NRGT), a plethora of ethical issues were raised in public discussions. Many of these were abstract, hypothetical and stirred by concerns and anxieties with a distinctly ‘western’ flavour. These concerns included questions of privacy, ownership, legitimacy, confidentiality and autonomy. In conversation with doctors and academics, however, three issues recurred in relation to IVF that were pressing, contentious and not easily ignored in the Sri Lankan setting. The first concerned sperm donation. Artificial insemination by husband was largely unproblematic in infertility treatments but the use of donor sperm was a source of major anxiety. The informal use of spermatozoa was known to be widespread and carried out with no records and minimal testing. In the absence of properly run and regulated sperm banks, spermatozoa were being procured from medical students, family members, casual acquaintances or doctors themselves in efforts to achieve a conception for a married couple (mostly using intrauterine injection). Anxieties about donor match, incest, future legal disputes over paternity and property and the liability of those carrying out such procedures were all rehearsed by those with whom I spoke. The overriding concern, however, was the possibility of human immunodeficiency virus/acquired immune deficiency syndrome (HIV/AIDS) transmission. At that time, the prevalence of HIV/AIDS in Sri Lanka was low. This was believed to be largely due to widespread condom use and the country's strong moral condemnation of promiscuity, prostitution, drug use and homosexuality. A new era in which IVF was firmly embraced would usher in new ways in which spermatozoa might be brought into circulation but also highlighted the need to bring old and potentially dangerous practices within the remit of new legislation. The second concern centred on the status of the embryo and the ways in which the accomplishment of IVF could result in embryos that are ancillary to requirements (e.g. in embryo reduction following multiple implantation). Whilst both Catholics and Buddhists marched in tune on the question of abortion, the issues raised by early stage destruction of embryos was far more complex to navigate. For Catholics in particular, the NRGT raised fundamental concerns about the destruction of ‘life’. For Buddhists, a rather different understanding of embryogenesis meant that early stage manipulation of gametes and embryos was, in theory at least, quite acceptable (Simpson, 2009). The third concern was the use of foreign expertise in IVF teams. Trained embryologists in particular were in short supply. This necessitated bringing foreign specialists in to fill the gaps in local knowledge. These bands of what might be thought of as IVF troubadours were, in the early 2000s, performing at great expense and with little regulatory oversight. As one IVF doctor put it: ‘they [foreign teams] can and come and go but it is me that has to face the music’. By this he was referring to the difficulties faced when dealing with the majority of parents for whom IVF does not result in a pregnancy, let alone a live birth. Indeed, unregulated and unchecked advertising by some clinics meant that client expectations of IVF were often wildly optimistic.

In short, there was broad agreement that regulation and legislation were needed to address these problems. Yet, the developments in question were mostly happening in a private sector in which innovation and enterprise did not sit easily with statutory regulation. In the meantime, IVF provision continued to grow, albeit in a regulatory vacuum.

## A first response: the NASTEC report

Following Professor Jayasekara's Genethics lecture there appears to have been a flurry of activity. The lecture was picked up by the National Science and Technology Commission (NASTEC), a body established by the Ministry of Science and Technology in 1998 to advise the Government of Sri Lanka on scientific policy. The head of NASTEC, Professor Noble Jayasuriya, issued an invitation to Professor Jayasekara to lead an ‘expert study group’ charged with developing a national policy on biomedical ethics. The six-person expert study group was, according to its chairman, specially selected to reflect not only technical expertise (two geneticists, a paediatrician, an obstetrician, a pharmacologist and a lawyer) but also a mix of men and women, married and unmarried, and different religious persuasions (Catholic, Buddhist and Hindu). While I was in Sri Lanka in 2000, the NASTEC committee was being convened and work was just beginning. At that time, the report was scheduled for publication in September of 2002.

When it appeared in 2003, the NASTEC report signalled a bifurcation in the direction of regulation. The structure of the report had two major sections: ‘Ethical Principles Relating to New Genetics’ and ‘Ethical Principles Relating to Assisted Reproductive Technologies’. Each section ended with a clearly signposted prescription for future regulation. There would be a new National Genetics Commission and an Assisted Reproductive Technologies Commission. The latter would be set up by an act of Parliament to be ‘the apex body overseeing the introduction and practice of assisted reproductive technologies both in research and in clinical settings in Sri Lanka …[]… Powers and the role of this commission should be similar to that of the Human Fertilization and Embryology Authority of the UK.’ (NASTEC, 2003: 29). This bifurcation had the effect of landing contentious issues about reproduction, gametes and embryos in one domain and those concerned with genetic medicine and diagnosis in another, thereby mapping out a future division of ethical labour.

Despite these lofty ideals, there was concern among some members of the Committee that where IVF was concerned the Ministry of Science and Technology was simply too preoccupied with more pressing issues to take much interest in the report. It was also felt, by contrast, that the Ministry of Health on the other hand, were likely to find the subject matter far too controversial. Without the guarantee of support from key government ministries, a different strategy would be needed. The idea that members of the NASTEC Expert Study Group had was that their efforts would translate first into guidelines and recommendations to go to the Sri Lanka Medical Council (SLMC). The SLMC would have to engage with the issue because of its statutory responsibility for overseeing foreign doctors operating in Sri Lanka and the fertility clinics were a place where they had a significant presence. With SLMC support in place, the Sri Lanka Medical Association and the College of Obstetricians and Gynaecologists would be brought in to help produce draft legislation. The timetable that was envisaged in 2003 would see an act of Parliament in approximately two years.

Following the publication of the NASTEC report, the initiative was indeed passed to the SLMC. The Ethics Committee of the SLMC drew on the technical expertise of Professor Jayasekara (Professor of Anatomy and former chair of the NASTEC Committee), Professor Harshalal Seneviratne (Professor of Obstetrics and Gynaecology at Colombo Medical Faculty who was also an IVF practitioner and Director of the Vindana Reproductive Health Clinic) and Dr. Malik Fernando (Chairman of the Ethics Committee of the SLMA). The SLMC would serve as the interim authority pending the establishment of a formal authority by an act of Parliament. As an interim authority, the SLMC published a Code of Practice in 2005 and required assisted reproductive treatment practitioners to register with the Council. Significantly, but not surprisingly given the SLMC's remit, the 2005 Code focused mainly on doctors, clinics and a voluntary code of practice. Unlike the NASTEC report, there was little in the Code of Practice that dealt with the complex ethical, legal and social issues that the assisted reproductive techniques bring in their wake, such as the status of gamete donors, surrogacy arrangements or anonymity. Guidance provided by the NASTEC report was thus in place but is little known outside specialist circles and is not legally binding. For example, surrogacy was dealt with in the NASTEC report as a solution for identified infertility problems. Formal adoption proceedings would be the only way to transfer legitimate parentage to the commissioning parents. In other words, the womb, not gametes, are given primacy. The NASTEC report clearly took the line that surrogacy should be treated as a ‘medical solution’ working in the interests of the nuclear family and, moreover, it should be non-commercial and could not be the subject of advertisements. The strictures around surrogacy might explain why some Sri Lankan parents reportedly source surrogate mothers in India, where the regulation of this sector has, in the past, been much more lax. More recently, there have been signs that individual Sri Lankan women have been advertising their services on international surrogacy matching websites. In short, much has been happening at the level of practice, but with the publication of the Code in 2005, the regulatory momentum stalled.

That bureaucratic machinery sometimes works exceedingly slowly and things get delayed is no different in Sri Lanka than anywhere else. In the bigger scheme of things, assisted reproductive treatment regulation in Sri Lanka was also not a particularly urgent priority given the state of the economy and the ongoing war. Moreover, those charged with drafting the documents were doing so as co-opted ‘volunteers’ who had to find time from their busy schedules for work that is exacting and likely to be contentious. These were plausible enough reasons when it came to explaining the ongoing regulatory vacuum. However, I would contend that the delay in regulation was not merely an absence of something but was a kind of presence that is worthy of analytical consideration.

Before that, however, let us consider for a moment what it is that is absent. Many of those with whom I spoke on the topic of IVF provision yearned for regulation that was public, state sanctioned and binding in its entirety, that is, regulation ‘with teeth’. This was what was felt to be needed to address the malpractice and unethical behaviour that they believed to be going on, particularly in some private sector clinics. There was disappointment that the country was failing in attempts to force a standardized legal and administrative order on a situation that was highly variegated and worryingly fluid and had been so from the outset – the sentinels were indeed slumbering. The situation prevailing in the sector seemed to sit somewhere between self-regulation (the presumption that practitioners are inherently decent people who will themselves refrain from acting unethically and take appropriate action if those around them do act in this way) and market regulation (the neo-liberal presumption that demand is the ultimate arbiter of service provision and the morality that goes with it). Against this backdrop, the work that goes into failing to produce regulation begins to be of considerable ethnographic interest, for it is not simply about tardiness, incompetence or self-interest, but points to a much deeper struggle around state, power and pluralism. Given the country's recent turbulent history, it is not surprising that regulation (here think ‘rule’, ‘order’, ‘force of law’, ‘a superior or competent authority’ and ‘control’) is not something that can be straightforwardly accomplished.

Nonetheless, the members of the expert study group were clear in discussion that their work was being undertaken for the ‘good of society’, for the ‘nation’ and to offer protection for values and morals that might be under threat from new technologies and that could ultimately cause people harm. Theirs was an endeavour to provide guidance in an area that needed regulatory oversight. The preface to the NASTEC report was signed off by the group's chairman with the hope that the report ‘would blossom into a document that is truly Sri Lankan’ (NASTEC, 2003:5). What is conveyed by these sentiments is an inclusive, democratic and tolerant vision of Sri Lanka as a secular nation state in control of technological progress, modernity and the future*.* Moreover, an important aspect of this vision is the capacity to come together in the face of external threats that might erode local values. The working party was thus explicitly and intentionally representative of religions and ethnicities but also avowedly non-partisan and secular in its operation and outputs. Yet, as Asad has argued, secularism references a ‘shallow’ universalism upon which claims to superiority over the divisive pluralisms of religion, culture and ethnicity are built (Asad, 2003). What lies beneath this shallowness is the fact that, whilst the intention might be to construct rights and ethics outside of religious identification, they are mostly lived through such denominations. This was particularly so in recent decades when the unified nation state almost failed to contain the plurality of visions of which it was made up. At that time, managing the paradox of secularism in public life required a heady blend of skill, creativity, diplomacy, guile and an ability to navigate a difficult and often dangerous social and political landscape. In assisted reproductive treatment, as in so many other attempts at public deliberation on contentious topics in Sri Lanka, the question of how to formulate a ‘national’ response and at the same time engage appropriately with religion was never far from the surface.

## The secular and the sacred in IVF regulation

In an important collection of essays, Bharadwaj and others illustrate the ways in which ideas of divine origins in human reproduction find their way, seemingly inexorably, into IVF practice (Bharadwaj, 2006). The collection demonstrates effectively how the relationship between science and religion is not one of immiscible layers but a complex blending in which patients and practitioners bring meanings to IVF practice that far exceed its technical specifications. What has been less well documented, and which I hope to throw light on here, is the same blurring of scientific and spiritual-cum-moral registers at the level of governance and regulation. At this level, it may be possible to discern how religious pluralism, to a greater or lesser extent, is safely accommodated within an apparently secular process of ethical deliberation.

As stated earlier, the expert study group on NGRT was made up of Hindu, Catholic and Buddhist representatives as a way of anticipating allegations of bias. Whilst for the Hindu community, interest in the new technologies was not a paramount concern, for Buddhists and Catholics it was. For practising Catholics, the field of assisted reproductive treatment poses challenges in a way that they do not for practising Buddhists. Buddhism is not a monotheistic religion and places a belief in rebirth determined by karma at its core. Christianity, however, views life as divine creation and, in theological terms, is far more likely to see interventions in the early stages of reproduction as in some way usurping God's will (Simpson et al., 2005).

Yet, as the majority religion, Buddhism is typically invoked as the backdrop within which other approaches are then accommodated (for example, see Fernando, 2014). Questions are thus raised as to whether Sri Lanka is a country that is home to multiple religious identities or one in which all other groups are simply subsumed under the hegemony of the dominant Sinhala Buddhist community (Krishna, 1999, Tiruchelvam, 2000, Wickramasinghe, 2007). The Catholic community is an interesting case in point. It is made up of both Sinhalese and Tamils who express allegiance to the Catholic Church. The community is closely attuned to the Vatican and the wider community of Catholics across the world as a source of guidance in matters spiritual and mundane. Catholics are well represented in the medical profession and have their own professional network, the Catholic Doctors' Guild of St. Luke and the Saints Cosmas and Damian (GOCD). At the time of my research they numbered between 300 and 400. Active members worked closely with Catholic leaders and laity, giving support and guidance, particularly in areas where Catholic teachings come into conflict with wider social practices and trends. Reproductive morality and the new technologies is a case in point. Over questions of abortion there is unanimity between Catholics and Buddhists. Over many other aspects of the NRGT there are disagreements of a more fundamental kind.

In August 2002 there was a papal delegation to Sri Lanka. One of the topics that the delegation wished to discuss with local doctors was the new technologies in Sri Lanka. The National Seminar on Bioethics and the Family was conducted by The Laity Council of the Catholic Bishops Conference of Sri Lanka and The Catholic Doctors Guild on 22–30 August 2002. The response to this event among Catholics with whom I spoke at the time was mixed. For some participants the tenor was mildly insulting. The Vatican view seemed to be neo-colonial in outlook and they expected to find people ‘still in trees and running round naked’ as one of them put it. It was suggested that the ‘line’ (on matters such as sexual permissiveness, contraception and abortion) had been lost in the West and the delegation was visiting the peripheries of the Catholic world to make sure it wasn't lost there. For others, the event was a vital reassertion of orthodox Catholic values in the face of incursions from IVF, stem cell research, sperm banks and AIDS, all of which opened the door to the desacralization of the embryo and abortion. An important document in establishing orthodoxy in this area is the 1968 *Humanae Vitae* of Pope Paul VI, which laid down clear but controversial guidelines on marriage, birth control and abortion. Significantly, section 24 makes reference to the pastoral responsibilities of scientists to preserve the relationship between ‘transmitting life’ and ‘married love’ (Vatican Encyclical, 1968). Among conservative elements of the Catholic medical profession these injunctions are taken very seriously and as a consequence the new technologies were, and continue to be, seen as a considerable threat. As one Catholic doctor described the emerging regulatory trend in Sri Lanka at the time: ‘there is no such thing as national consensus or national regulation. The majority get their way but that doesn't make it ethical or moral’. What this particular doctor was trying to get across was his frustration at the absence of regulation and, moreover, the way in which all sorts of practices appeared to be sliding into place and about which, as a strong defender of Catholic faith and principles, he was deeply unhappy. In a reprise of the very familiar ‘slippery-slope’ argument, the possibilities of assisted reproductive treatment, if unchecked, would undermine the very foundations of the conjugal family. Use of donor gametes, surrogacy, casual disposal of embryos, embryos for research, the possibility of reproduction in which neither men nor marriage seem to figure and much else, shocked and perplexed him in equal measure. In looking to the West he could see all too clearly what the likely consequences of an unchecked embrace of assisted reproductive treatments would be and what the country needed to be protected from. Once again, the sleeping sentinels theme resonates. Values and traditions have to be protected by people like him because the ‘majority’ have little sense of their worth and they do not understand how they are so easily lost. The sanctity of the family for Catholics is perhaps the most emblematic of these concerns. However, beneath these concerns lay a more general unease with the ways in which moral accountability is reckoned between members of different religious communities. Lurking in the reference to ‘the majority’ was the majority Buddhist community and the notion that they were less motivated to act over such issues than Catholics. His perception was of Buddhists as generally interested in little beyond their personal karmic accounting and therefore less likely to check what is going on in the next person's. In this climate, as he saw it, an easy and dangerous permissiveness was all too easily fostered.

This doctor's concerns raise a more fundamental question of representation in deliberative processes such as expert reviews, working parties, standing committees and other mechanisms for ensuring that the democratization of scientific progress works in plural settings. Among those who were broadly connected with the development of bioethics and governance in Sri Lanka at that time, it was generally acknowledged that there should be community representation in the regulation of the new technologies*.* However, ‘community’, typically equates with representation from major religious groupings and there were often misgivings about how to effect such a strategy. It was felt that involvement could bring the divisive and destructive assertion of religious fundamentalisms into play. There were many who would be quick to pronounce innovation in technology, ethics and regulation, as anti-Buddhist, anti-Christian or anti- any other of the denominations active in Sri Lanka, and particularly so if these innovations emanate from the West. In the process of consultation, therefore, the trick would appear to be one of constructing frameworks that are ‘secular’ enough to allow rational dialogue to proceed, but representative enough for reassurance that diverse communities have a channel to express their voice. Speaking of the make-up of a future regulatory authority, Seneviratne puts it thus: ‘It is therefore necessary that those institutions established to rule on such issues when they arise should consist of members who are technologically competent, are aware of the social and religious sensitivities of the country, knowledgeable of the law of the land, and have the maturity to deal with such situations’ (Seneviratne, 2011:81). I assume that what is meant by ‘maturity’ here is the ability to handle the considerable pressure on those who find themselves not only as representatives of expertise but also as the signifiers of others' interests.

In Sri Lanka, a person's identification with one religious community or another is something that is likely to be learned at an early stage in getting to know them. It may be disclosed directly as part of the ‘presentation of self in everyday life’, inferred indirectly by a person's references or actions or disclosed by a third party. Among the doctors, academics and clinicians with whom I worked I mostly knew which faith they professed (or in some cases had stopped professing). However, knowing a person's public religious persona says little about what they actually profess or practice in private. This distinction is important. In a country such as Sri Lanka, where religiosity is so apparent and so pervasive, it is not just the relationship between publicly held views and private religion that is of interest to others. For those in public life, the relationship between privately held views and public religion is also important. In other words, taking on responsibility for acts that articulate a collective or ‘common’ sense necessarily entails an acknowledgement of other positions, religious and non-religious, as well as having visibility and credibility within one's own community.

In Sri Lanka, the importance of this referencing across communities whilst referencing back to one's own is considerably amplified given the densely interwoven patterns of social relationships and shared personal and professional history within the medical profession. As was often pointed out, an advantage of working in Sri Lanka is the ‘small world’ in which people operate, socially and professionally. The flip-side of this, however, is evident in the concerns about the extent to which nepotism and ‘cronyism’ and undue political interference might shape decision-making processes. The smallness of worlds also tends to mean that, in series or at the same time, people might wear the hats of regulator, government advisor, private practitioner. This is not to suggest that there is duplicity but rather that the assisted reproductive treatment sector is very small by comparison with those of other countries, and consequently expertise is drawn from a small pool. Influence over the private sector was a particular source of anxiety in this regard and there was some despondency among those charged with responsibility for regulation that honest attempts to realize procedures that are fair, transparent and robust are all too often confounded as decisions made or guidelines agreed meet with limited compliance outside of this or that committee. The history of IVF regulation is a case in point, with some doctors expressing doubts as to whether, as long as the private sector was so much in the ascendant, any meaningful regulation of the new technologies was possible at all.

## Conclusion

On my last visit to Sri Lanka in December 2014, the draft of the Bill regulating assisted reproductive treatment was almost, but still not quite, finished. The National Bioethics Council (NBC) in collaboration with the Sri Lanka Medical Council had set about drafting this legislation in 2006 in the form of a Human Reproduction and Genetics Act (HURGA) (Fernando, 2014:1522). The fields of genetics and reproduction had once again been brought together. The principal aim of the act was now to establish a Human Reproduction and Genetics Authority for Sri Lanka. What had been earlier separated by one group looked set to be reunited by another.

In as much as there was regulation in 2014, this still lay with the SLMC through its requirements for practitioner registration and adherence to the 2005 voluntary Code of Practice. The number of clinics in Colombo, the capital city, had grown to seven, with two further clinics operating in provincial towns and more on the way. The number of IVF children born in Sri Lanka had risen to several thousand, although accurate numbers are impossible to ascertain. There were still hopes that a state hospital offering free infertility services might be established. Whilst such a facility would carry out assessments, offer access to donor spermatozoa and intrauterine insemination and advise on surrogacy arrangements, state-funded IVF was likely to be a long way off given the costs and the shortage of infertility specialists in the public sector. The private sector remained the primary supplier of services. Consequently, concerns about the three anxieties identified at the beginning of this account remained high. These were: (i) the unregulated use of spermatozoa and the association with HIV and also its consequences for normative models of legitimacy and family; (ii) the fate of the embryo in IVF practices; and (iii) the commercial traffic in people, doctors and biological materials in and out of Sri Lanka, and particularly to and from India. All of these remained problematic concerns for the SLMC. Adding to these were now concerns that some practitioners were acting unethically by recommending IVF as a solution to infertility long before other easier, cheaper and less invasive routes had been exhausted and, moreover, making wild claims for their success rates. At the time of writing, the call for legislation among practitioners is as strong as ever (Palihawadana and Seneviratne, 2015, Seneviratne, 2011). Legislation is edging its way through the Ministry of Health and then past their legal draughtsman before it goes to ministers to be passed in Parliament. Until this happens, however, a climate of ‘soft’ regulation continues and with it an ethical fluidity in which multiple and often contradictory religious perspectives on the assisted nb resproductive treatments can remain, more or less, safely in play (*cf* Clarke, 2015 who makes a similar case for assisted reproductive treatment in Lebanon). Rather like an encounter with a Möbius strip, participants appear to traverse a continuous surface without ever crossing an ‘edge’. Under such conditions, the commercial sector and the services it offers continue to develop untrammelled either by legislation and the oversight of ‘a superior or competent authority’ or by the moral self-constraints of service users.
